# Treatment and survival analysis for 40-year SEER data on upper esophageal cancer

**DOI:** 10.3389/fmed.2023.1128766

**Published:** 2023-07-17

**Authors:** Xi Wu, Ming-Chuang Zhu, Guo-Liang Li, Peng Xiong, Wei Sun, Ni Zhang, Bo Zhao, Le-Qun Li, Xiang-Ning Fu, Min Zhu

**Affiliations:** ^1^Department of Thoracic Surgery, Tongji Hospital, Tongji Medical College, Huazhong University of Science and Technology, Wuhan, China; ^2^Shanxi Province Cancer Hospital/Shanxi Hospital Affiliated to Cancer Hospital, Chinese Academy of Medical Sciences/Cancer Hospital Affiliated to Shanxi Medical University, Taiyuan, China; ^3^Department of Radiation Oncology, The Affiliated Hospital of Qingdao University, Qingdao, Shandong, China; ^4^Intensive Care Unit, The Central Hospital of Wuhan, Tongji Medical College, Huazhong University of Science and Technology, Wuhan, China

**Keywords:** upper esophageal cancer, long-term survival, surgery, radiotherapy, the surveillance, epidemiology, end results database

## Abstract

**Background:**

Upper esophageal cancer (UEC) is rare in both Eastern and Western countries. The epidemiological characteristics and long-term survival of UEC patients are less known. In addition, the choice of optimal treatment for UEC has been controversial.

**Methods:**

Cases of UEC (C15.3 and C15.0) arising during the period from 1973 to 2013 were identified and selected using the SEER database. Student's *t*-test and Pearson's chi-square test were used to compare the differences in parameters among different groups. Esophageal cancer-specific survival (ECSS) and overall survival (OS) rates were calculated by using the Kaplan–Meier method. Cox proportional hazard regression was used to analyze predictive factors.

**Results:**

In the past 40 years, the cases of UEC have gradually increased, and the proportion of adenocarcinoma (AD) has gradually increased (from 3.6% to 11.8%, *p* < 0.001). There has been a significant increase (1973–1982 vs. 2004–2013) in median OS (7 months vs. 10 months, *p* < 0.001) and median ECSS (7 months vs. 11 months, *p* < 0.001) among UEC patients from 1973 to 2013. For the impact of different treatments, the results showed that the ECSS and OS of surgery without radiation (SWR) and radiation plus surgery (R+S) were superior to those of radiation without surgery (RWS). Subgroup analysis showed that ECSS and OS were highest among patients treated with SWR compared with R+S and RWS for patients with localized disease. For regional disease, ECSS and OS were highest among patients with R+S compared with SWR or RWS. Among patients with regional-stage squamous cell carcinoma (SCC), OS was higher with neoadjuvant radiotherapy or adjuvant radiotherapy compared with SWR. Multivariate analysis showed that radiotherapy sequence was dependently associated with OS among patients with regional-stage SCC.

**Conclusion:**

Although the long-term survival of UEC remains poor, it has gradually increased since 1973. This should be closely related to the improvement of medical care over the past 40 years. Different treatment methods have a great influence on the long-term survival of UEC. For localized diseases, surgery may be a better choice. For regional disease, surgery plus adjuvant or neoadjuvant radiotherapy may be more beneficial to improve the long-term prognosis of UEC patients.

## Introduction

Esophageal cancer is one of the most common malignant tumors in the world, ranking sixth in morbidity and eighth in mortality (1). Esophageal cancer rarely affects the upper esophagus, including the cervical and upper thoracic esophagus, which accounts for only 5–10% of all cases of esophageal cancer (2–4). Compared with carcinoma affecting the middle or lower segments of the esophagus, carcinoma of the upper esophagus is challenging. Multidisciplinary treatment is always required because of the complicated anatomy of the upper esophagus, and because carcinoma affecting the upper esophagus is typically advanced at the time of diagnosis, with a tendency to invade surrounding anatomical structures when being diagnosed (3–6). Thus, UEC is associated with a poorer prognosis than any other type of esophageal cancer (6).

In general, treatment of UEC includes surgery, radiotherapy (RT), chemotherapy, or a combination of these approaches. Surgery was once the major management of UEC. The surgical method used most commonly by UEC is the McKeown approach (tri-incisional esophagectomy) (7), which always requires cervical or total esophagectomy. A pharyngo-laryngo-esophagectomy (PLE) is required in the case of a high disease burden (5). Therefore, postoperative complications are common, and the 5-year overall survival (OS) for surgical resection is low (12–33%) (8, 9).

Previous reports have found similar OS after surgery, radiotherapy (RT), and definitive chemoraidotherapy (CRT) (5, 10). RT and CRT gradually became the preferred treatments for UEC in many countries and regions, including the United States (3, 11, 12). However, among patients with resectable tumors, long-term outcomes were significantly improved for those who underwent surgery compared with those who received only received definitive CRT (13). Surgery may also result in improvements in prognosis, quality of life, and post-treatment dysphagia symptoms (10, 14). The centers included in these studies continue to rely on surgery as the primary treatment for UEC (5, 8, 13).

The surgical procedure for esophageal cancer has changed dramatically in recent years with the development of medical skills and instruments. For example, many centers have adopted minimally invasive esophagectomy. The advantages of minimally invasive esophagectomy compared with open esophagectomy include decreased postoperative pain, decreased length of hospital stay, and fewer complications (15, 16). Furthermore, surgical robots have been used widely to perform esophageal cancer operations, with positive clinical outcomes (17, 18). Technological advancement has also brought numerous improvements to RT and chemotherapy (2, 12).

However, esophageal carcinoma of the upper segment is a rare type of esophageal cancer. Due to its small number of cases, it accounts for a very small proportion of clinical trials of esophageal cancer compared with middle- and lower-segment esophageal cancer. In addition, clinical studies on upper esophageal carcinoma are relatively lacking (19). Upper esophageal cancer is not well understood, and the choice of treatment for upper esophageal carcinoma is still controversial.

The National Cancer Institute's Surveillance, Epidemiology, and End Results (SEER) database is a cancer database covering ~26% of the total US population. This database includes information on the incidence of cancer in 18 areas of the US (20). This study aimed to use information from the SEER database to analyze the epidemiological characteristics and long-term survival trends of UEC over the past 40 years. In addition, the effects of different treatment strategies on the survival of patients with UEC were also analyzed to make people have a clearer and deeper understanding of UEC.

## Methods

### Database and patients

This study was performed using data from the SEER database. Data were collected during the period from 1973 to 2013 (available at: www.seer.cancer.gov) based on the November 2015 submission using SEER^*^Stat software, version 8.3.5.

The outcomes of interest in this study were OS and esophageal cancer-specific survival (ECSS), according to specific codes. We collected information for patients with UEC diagnosed during the period from 1973 to 2013. All patients included in the study had a primary site-labeled recode diagnosis of “C15.0- Cervical esophagus and C15.3-Upper third of esophagus” (from the lower margin of the sixth cervical vertebra to the superior margin of the sixth thoracic vertebrae). Exclusion criteria were multiple primary carcinomas, an unknown number of survival months, and diagnosis < 1 month prior to death. Tumors were classified as squamous cell carcinoma (SCC) (8050-8082), adenocarcinoma (AC) (8140-8573), or “other” pathological type. Criteria for SEER historical stage A (localized, regional, and distant) were adopted in order to comply with a unified tumor staging system across all years of the study.

### Statistical analysis

Continuous variables were compared with Student's t-test; categorical variables were compared with Pearson's chi-square test. The Kaplan–Meier (KM) methods were used to estimate survival time for time-to-event endpoints, OS, and ECSS. The log-rank test and Cox proportional hazards models were used to conduct univariate and multivariate analyses, respectively. Multivariate analysis included only those variables that were significantly associated with survival in univariate analysis. All p-values were two-sided, and *p* < 0.05 was considered statistically significant. All analyses were performed using IBM SPSS version 20.0.

## Results

### Patient characteristics

The study flow chart is shown in Supplementary Figure 1. A total of 4424 patients who met our inclusion criteria were included in the study (Supplementary Table 1). The mean age was 66.8 years. Men accounted for 66.5% of all patients. Most patients had regional (37.6%) or distant (31.4%) stages of disease at the time of diagnosis. First, patients were divided into four groups according to the year of diagnosis (Supplementary Table 2). Although SCC (85.3%) remained the most common pathological type of UEC, the proportion of AC gradually increased over the study period (from 3.6% to 11.8%, *p* < 0.001). Most patients had undergone RT (75.7%) and only a few (13.3%) had been treated with surgery. While patients with localized or regional-stage diseases were more likely to receive surgery (*p* < 0.001). RT was more commonly used in patients with regional-stage disease (*p* < 0.001 Supplementary Table 3). The rate of surgical resection was higher for AC compared with SCC. Patients with SCC were more likely to receive RT than patients with AC (*p* < 0.001 for all, Supplementary Table 4).

### Treatments

Patients were divided into four groups based on the type of treatment received (Supplementary Table 5). Patients with localized diseases were more likely to undergo SWR, while patients with regional-stage tumors tended to choose R+S (p < 0.001). Patients who chose neither surgery nor radiotherapy tended to have advanced-stage diseases (*p* < 0.001).

In order to evaluate the effect of RT sequence on survival among patients who underwent surgery, we divided patients into three groups (Supplementary Table 6): SWR, neoadjuvant radiotherapy (NRT), and adjuvant radiotherapy (ART). The proportion of SCC was lower in the SWR group compared with the ART and NRT (*p* < 0.001). The number of patients who elect to undergo NRT has increased over recent decades (*p* < 0.001). SWR was most likely to be performed for patients with localized disease (*p* < 0.001). While ART was most commonly used in the treatment of patients with regional stage (*p* < 0.001), NRT was most commonly used in patients with distant stage (*p* < 0.001).

### Patient survival

#### Overall

Median OS was ~9.0 months (95% CI: 8.65–9.35). Overall, ECSS was also ~9.0 months (95% CI: 8.60–9.40). OS at 1, 3, and 5 years was 37.0%, 13.2%, and 10.0%, respectively; ECSS at 1, 3, and 5 years was 40.8%, 17.9%, and 14.1%, respectively.

**Figure 1:**
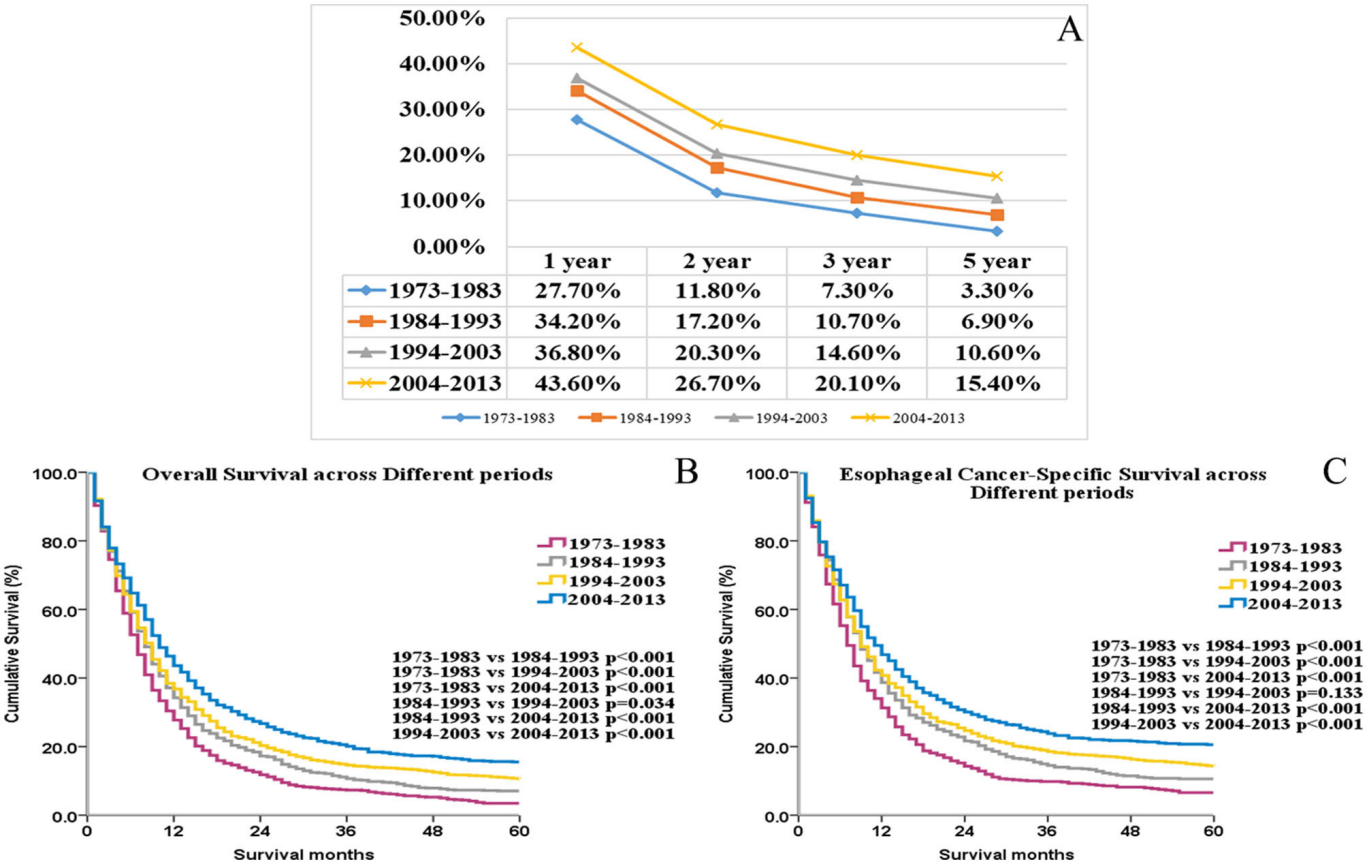
The trends of the 1-, 2-, 3-, and 5-year overall survival rate for UEC patients **(A)**. The OS and ECSS of UEC patients across different periods **(B, C)**.

Overall survival values at 1, 2, 3, and 5 years, respectively, increased further for each year that elapsed between 1973 and the time of diagnosis (Figure 1A). OS and ECSS differed significantly among these four groups (*p* < 0.05 for all, Figures 1B, C). These results indicate significant increases (1973–1982 vs. 2004–2013) in median OS (7 months vs. 10 months, *p* < 0.001) and median ECSS (7 months vs. 11 months, *p* < 0.001) since 1973.

**Figure 2:**
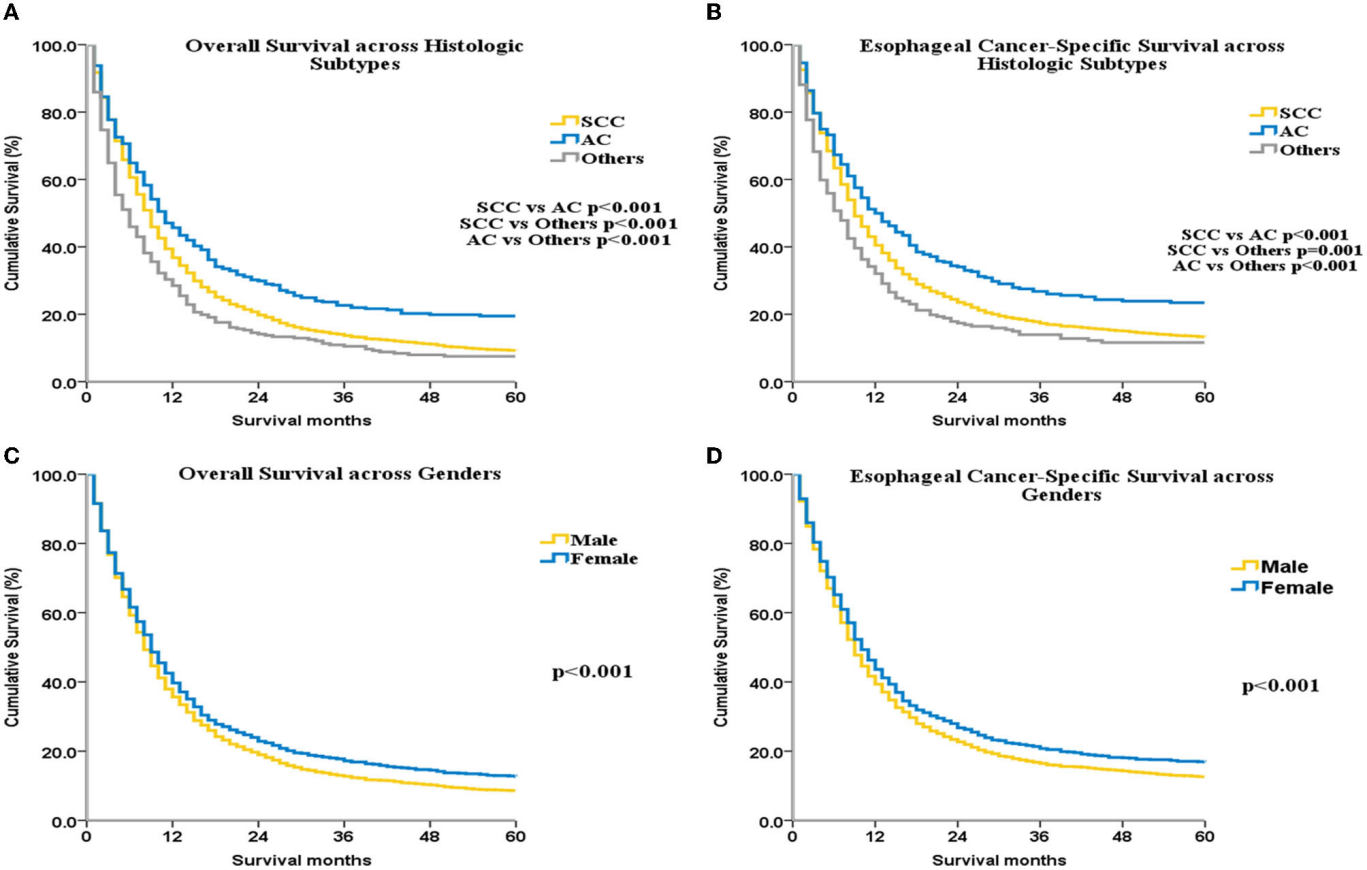
OS and ECSS for UEC patients across different histological subtypes **(A, B)** and different genders **(C, D)**.

The OS and ECSS were greater for AC than for SCC (*p* < 0.001 for all, Figures 2A, B). OS was higher among women compared with men (*p* < 0.001; Figure 2C). ECSS was also higher among women compared with men (*p* < 0.001; Figure 2D). Univariate (Supplementary Table 7) and multivariate (Supplementary Table 8) Cox analyses identified the following independent factors associated with ECSS as well as OS: date of diagnosis, ethnicity, sex, age, marital status, histologic subtype, SEER historic stage, surgical treatment, and RT.

### Survival analysis among different treatment groups

**Figure 3:**
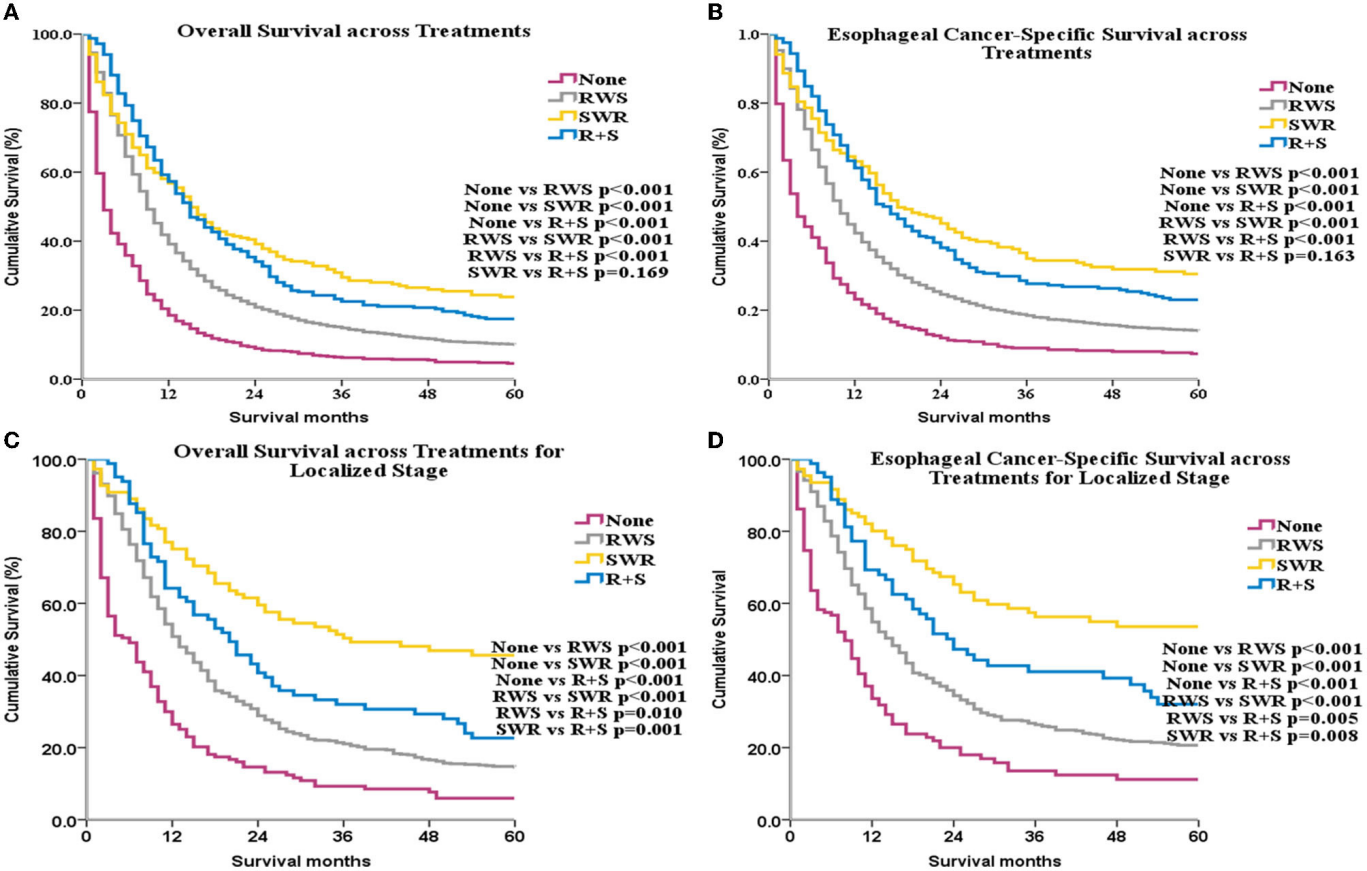
OS and ECSS across different treatments for all patients **(A, B)** and for patients with localized stage disease **(C, D)**.

Median OS for the control, RWS, SWR, and R+S groups was 3 months, 9 months, 15 months, and 15 months, respectively. ECSS and OS were improved among patients who underwent RT or surgery compared with patients who did not receive treatment (*p* < 0.001 for all, Figures 3A, B). ECSS and OS were lower in the RWS group compared with the SWR and R+S groups (*p* < 0.001 for all, Figures 3A, B).

**Figure 4:**
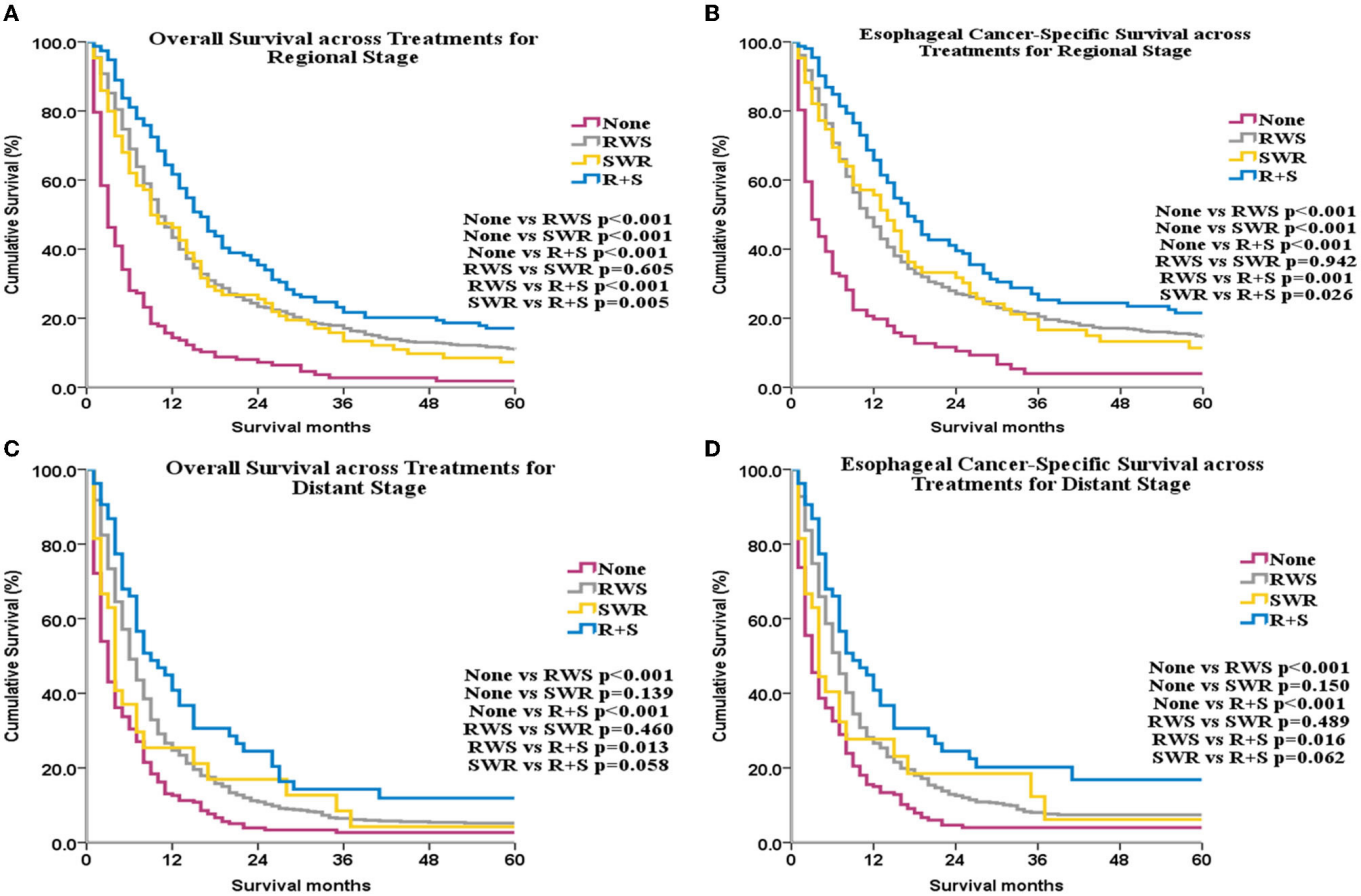
OS and ECSS across different treatments for patients with regional stage disease **(A, B)** and for patients with distant stage disease **(C, D)**.

Subgroup analyses by SEER historical stage A revealed that, for patients with localized disease, ECSS and OS were greatest in the SWR group (Figures 3C, D). For patients with regional disease, ECSS and OS were highest in the R+S group (Figures 4A, B). Univariate (Supplementary Table 9) and multivariate (Table 1) analyses demonstrated that treatment strategy was independently associated with both ECSS and OS. For patients with distant stage disease, OS and ECSS were higher than None and RWS group (*p* < 0.05, Figures 4C, D). However, the fluctuation of OS and ECSS curves are relatively large in SWR group for patients with distant stage disease (Figures 4C, D).

**Table 1:** Multivariate analysis of ECSS and OS across treatments.

**Variable**	**ECSS**			**OS**		
	**HR**	**95% CI of HR**	***P*** **-value**	**HR**	**95% CI of HR**	***P*** **-value**
**Group**				
None	Reference			Reference		
RWS	0.514	0.468–0.564	< 0.001	0.501	0.459–0.547	< 0.001
SWR	0.380	0.316–0.457	< 0.001	0.383	0.324–0.452	< 0.001
R+S	0.362	0.309–0.424	< 0.001	0.367	0.317–0.424	< 0.001
**Year of diagnosis**				
1973–1983	Reference			Reference		
1984–1993	0.773	0.688–0.869	< 0.001	0.789	0.709–0.879	< 0.001
1994~2003	0.665	0.596–0.742	< 0.001	0.662	0.597–0.733	< 0.001
2004~2013	0.485	0.435–0.542	< 0.001	0.488	0.440–0.541	< 0.001
**Ethnicity**				
White	Reference			Reference		
Black	1.160	1.064–1.266	0.001	1.165	1.074–1.264	< 0.001
Other	0.997	0.877–1.133	0.959	0.969	0.859–1.093	0.607
Unknown	1.419	0.734–2.742	0.298	1.260	0.652–2.432	0.492
**Sex (Male)**				
Male	Reference			Reference		
Female	0.845	0.782–0.913	< 0.001	0.843	0.784–0.906	< 0.001
**Age**				
≤ 45	Reference			Reference		
>45, ≤ 65	1.576	1.262–1.968	< 0.001	1.669	1.351–2.061	< 0.001
>65, ≤ 80	1.619	1.296–2.024	< 0.001	1.805	1.460–2.231	< 0.001
>80	1.878	1.473–2.393	< 0.001	2.172	1.728–2.731	< 0.001
**Marital status**				
Married	Reference			Reference		
Single (unmarried)	1.138	1.027–1.261	0.013	1.128	1.025–1.242	0.014
Separated/divorced/widowed	1.148	1.057–1.248	0.001	1.123	1.039–1.213	0.003
Unknown	1.140	0.947–1.372	0.167	1.064	0.892–1.268	0.492
**SEER historic stage**				
Localized	Reference			Reference		
Regional	1.522	1.378–1.681	< 0.001	1.434	1.310–1.571	< 0.001
Distant	2.279	2.054–2.529	< 0.001	2.064	1.874–2.273	< 0.001
Un-staged	1.249	1.117–1.398	< 0.001	1.204	1.087–1.333	< 0.001

### Radiation sequence with surgery

**Figure 5:**
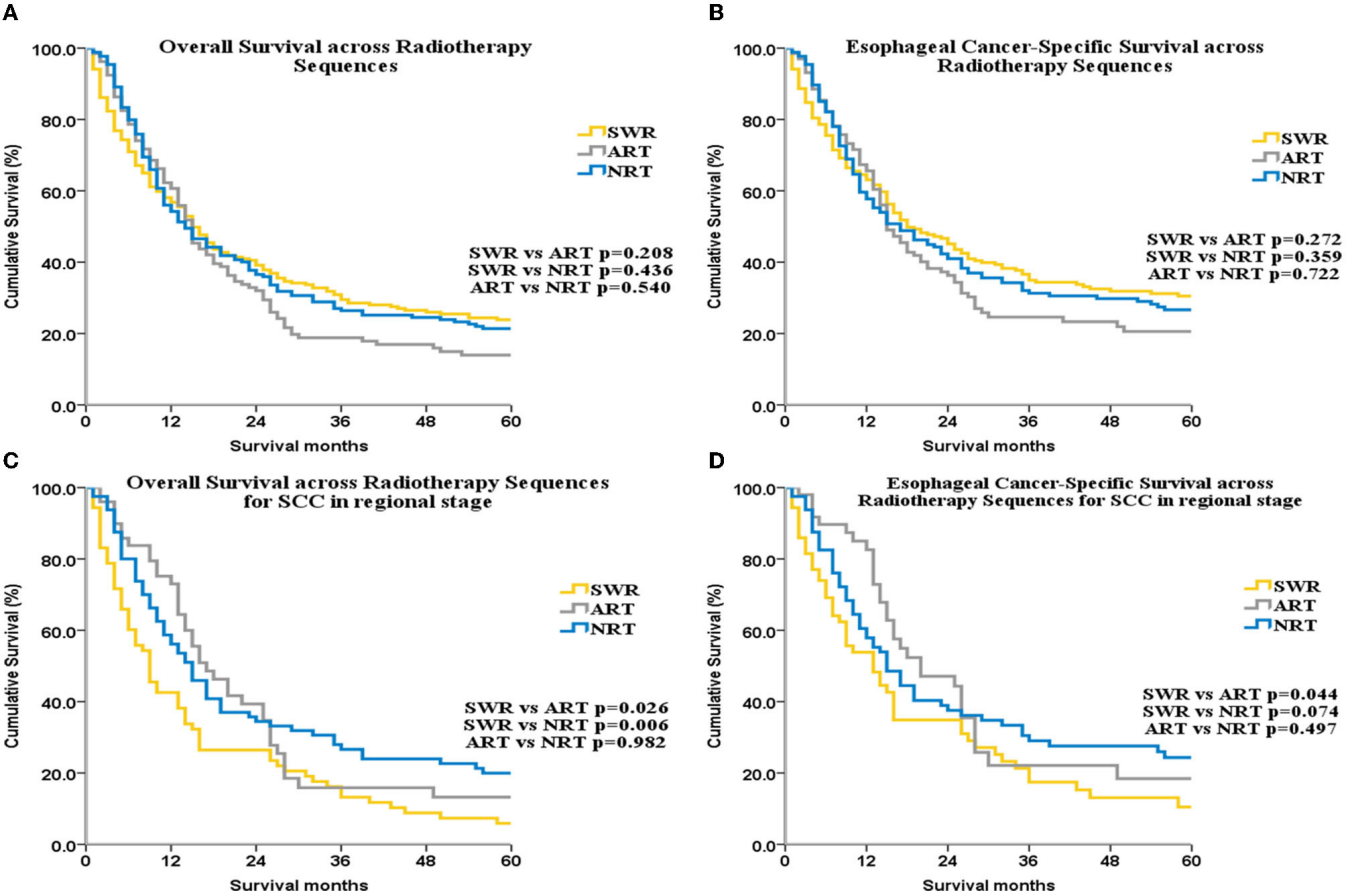
OS and ECSS across radiotherapy sequences for all patients **(A, B)** and for patients with SCC patients with regional stage disease **(C, D)**.

No significant difference in ECSS or OS was found among the SWR, NRT, and ART groups (*p* > 0.05 for all, Figures 5A, B).

Sub-group analysis by SEER stage showed that, for patients with localized disease, OS was highest in the SWR group. Among patients with regional disease, OS was lowest in the SWR group compared with the ART and NRT groups. However, multivariate analysis did not reveal a significant difference in OS in localized or regional sub-groups.

Next, we performed sub-group analysis by histologic subtype. For the SCC subgroup, among patients with regional disease, OS was lower with SWR (median OS: 9 months, 95% CI: 6.34–11.67) compared with NRT (median OS: 17 months, 95% CI: 11.79–22.21) and ART (median OS: 15 months, 95% CI: 11.42–18.59; Figure 5C). Multivariate analysis for this subgroup also demonstrated that RT sequence was an independent factor for OS (SWR as reference, HR of NRT: 0.633, 95% CI: 0.427–0.938, *p* = 0.023; HR of ART: 0.635, 95% CI: 0.453–0.889, *p* = 0.008, Supplementary Table 10). No other subgroup analysis yielded statistically significant results. ECSS was higher in the ART group compared with SWR group for SCC patients with regional disease (*p* = 0.044, Figure 5D). There were no significant difference for ECSS between ART group and NRT group (*p* = 0.479, Figure 5D). Although the trend of ECSS in ART group was better than that in SWR group, it was not statistically significant (*p* = 0.074, Figure 5D).

### Comment

Although the long-term survival of patients with UEC remains extremely low, this figure gradually increased in the US during the period from 1973 to 2013. This trend may reflect advancements in medical equipment, surgical technique, and related adjuvant therapy. OS and ECSS were higher among women compared with men, perhaps due to gender-based differences in lifestyle. The use of alcohol and cigarette smoking is more common among men, and both are common causes of esophageal cancer (1).

Recent decades have also seen a dramatic rise in the incidence of esophageal adenocarcinoma (EAC) in Western countries (21, 22). The results of our study indicate a similar rise in the prevalence of UEC. Notably, AC was rarely found in the upper esophagus (23, 24). And the histogenesis of AC in the upper esophagus remains unclear. The pathogenesis of AC in the middle and/or lower esophagus is typically related to gastroesophageal reflux or Barrett's esophagus. However, the pathogenesis of AC in the upper esophagus is different from that of AC in the middle or lower esophagus. Previous studies have revealed that AC in the upper esophagus may arise from esophageal glands or heterotopic gastric mucosa (HGM), the latter of which may be related to infection with helicobacter pylori (24). Our results presented above identified histologic type as an independent prognostic factor in UEC. Survival was higher for AC than for SCC, as reported previously (25). Among patients with regional or distant disease, SCC was more common than AC (Supplementary Table 4). This finding is concordant with the results described above, which found that lymph node metastasis was more commonly associated with SCC than with AC (25).

In most countries and regions including the US, first-line strategies for UEC are CRT and RT (2, 3, 11, 12). Our study revealed that RT was commonly used. For SCC, this proportion reached 70.6%. Previous studies have reported no significant difference in outcomes after combined surgery and RT compared with CRT alone (5). However, other studies have shown that among patients with resectable UEC, long-term outcomes appear to be better with surgery plus CRT compared with definitive CRT alone. Patients who underwent surgery also had improvements in prognosis, quality of life, and post-treatment dysphagia (10, 14). In our study, OS and ECSS were higher among the SWR and R+S groups compared with the group of patients who underwent RT alone. Among patients with localized disease, survival was highest with surgery alone compared with RWS and R+S. Notably, RT may decrease the patient's autoimmunity and increase the risk for postoperative complications (26, 27). Furthermore, the use of RT alone was likely insufficient to entirely eliminate the primary lesion. For patients with regional disease, the highest OS was found in the R+S group. This finding is similar to those published in a previous report (13). Importantly, R+S, in addition to removing the lesion, eliminated any potential unresected lesions.

Surgery may afford reasonable OS and improve the quality of life for UEC patients (28). Surgery may also significantly reduce local tumor recurrence and improve dysphagia (14). The surgical method used most commonly to treat UEC is the McKeown approach (tri-incisional esophagectomy) (7). Surgery is not recommended for tumors < 20 cm from the incisors, because UEC at this site always requires a pharyngo-laryngo-esophagectomy, which is associated with poor quality of life post-operatively because of the loss of vocal function (29). Previous studies also showed that long-term survival was similar in the CRT and surgery groups for patients with lesions that were positioned more superiorly (14, 30). Thus, CRT gradually became the main strategy for these patients. However, the prognosis for these UEC patients who underwent definitive CRT alone was always unsatisfactory because of insufficient local disease control; salvage surgery was always needed for these patients (29). In recent years, studies have sought to elaborate on a larynx-preserving surgery that would preserve vocal function in patients with UEC (29). Furthermore, larynx-preserving surgery compared with non-preserving procedures was associated with improved prognosis and decreased complications (29). However, a comparison of larynx-preserving surgery and CRT alone showed no significant difference in long-term survival (28). Compared with CRT alone, surgery decreases the risk of local recurrence and improves dysphagia symptoms (14). Therefore, larynx-preserving surgery may be an acceptable surgical approach for UEC proximal to the larynx.

Nowadays, the combination of surgery and chemoradiotherapy or immunotherapy is increasingly preferred for the treatment of resectable esophageal cancer (31, 32). Clinical practice has recently seen the increased use of adjuvant and neoadjuvant therapies for the treatment of esophageal cancer. However, the role of adjuvant and neoadjuvant therapies remains controversial. For neoadjuvant radiotherapy (NRT), previous studies reported that NRT compared with surgery alone, may improve 5-year OS and increase the curative resectability of tumors (33). However, other researchers found no increase in resectable rate or OS among patients treated with NRT (34). For neoadjuvant chemotherapy (NCT), some researchers believed that patients were more tolerant to chemotherapy response before the operation. Ando N et al. found that the 5-year OS rate was 55.00% in the NCT group and 43.00% in the postoperative chemotherapy group (*P* = 0.04) of 330 patients with stage II/III (35). Another study showed that the NCT group had a better R0 removal rate (60.00% vs. 50.00%) and a better 5-year OS (23.00% vs. 17.10%, HR = 0.84; 95% CI: 0.72 ~0.98, *P* = 0.003) compared with surgery alone (36). The other study also showed that NCT can improve the radical resection rate, PFS, and OS of patients with esophageal adenocarcinoma (37). However, the study results of Kelsen et al. (38) showed that NCT could not improve postoperative OS in patients with locally advanced esophageal cancer, but patients with tumor response in the NCT group had improved OS. This study also found that only R0 resection could bring significant survival benefits. There was no significant difference in the median OS among R1, R2, and unresected patients (38). Patients receiving neoadjuvant chemoradiotherapy (NCRT) had a higher pathological complete response (pCR) rate and greatly improve the tumor resection rate. Compared with surgery alone, patients who received NCRT plus surgery had better OS and PFS. In addition, the R0 rate was also higher than surgery alone (39–41).

In addition, several studies are ongoing or have shown results regarding the role of immunotherapy as adjuvant or neoadjuvant therapy for esophageal cancer (42, 43). Notably, none of these studies studied UEC alone. In our study, NRT was mainly used in patients with advanced disease (Supplementary Table 5, *p* < 0.001), suggesting that NRT may improve tumor resection rate. However, for patients undergoing surgery, there was no significant effect of NRT or ART on OS or ECSS (*p* > 0.05 for all, Figures 5A, B). OS for patients with regional disease was higher in the ART and NRT groups compared with the SWR group. This finding was expected because lymph node metastasis is observed in almost all patients with regional disease. NRT and ART may eliminate potentially metastatic lesions and, thus, reduce tumor recurrence.

The radiotherapy sequence was not an independent factor in the NRT and ART subgroups. In a previous analysis of the SEER database, NRT was beneficial to long-term survival and an independent factor for OS (33). However, this previous analysis did not study UEC alone. In our study, which did investigate UEC alone, RT sequence was an independent factor for OS among patients with regional SCC.

Current treatment for UEC typically includes CRT or surgery plus CRT. However, we could not assess the effect of chemotherapy on patent survival, as these data were not available in the SEER database. RT methods, radiation dose, and surgical methodology are also reported to have significant effects on patient prognosis (2, 12, 15). However, the SEER database does not include these data. The database did not register information related to smoking, drinking, or postoperative complications. Finally, we used SEER historical stage A (localized: confined to the primary site; regional: spread to regional lymph nodes; distant: cancer metastasis) in order to comply with unified tumor staging criteria across all years of the study. SEER historical stage A differs from the staging criteria provided by the American Joint Committee on Cancer (4). Any of the factors mentioned above may have resulted in a deviation in the research results.

## Conclusion

Although the long-term survival of UEC patients remains poor, it has gradually increased from 1973 to 2013. This should be closely related to the improvement of medical care over the past 40 years. Our results also showed that different treatment methods have a great influence on the long-term survival of UEC patients. For patients with localized disease, surgery may be a better choice. For patients with regional disease, surgery plus adjuvant or neoadjuvant radiotherapy may be more beneficial to improve the long-term prognosis of UEC patients. Additional clinical studies are needed to identify the optimal treatment strategies for UEC.

## Data availability statement

The original contributions presented in the study are included in the article/Supplementary material, further inquiries can be directed to the corresponding author.

## Author contributions

MZ conceived, designed, and supervised the study. XW, M-CZ, and G-LL collected, analyzed, visualized the data, and wrote the draft of the manuscript. PX, WS, and NZ provided support for the statistical analysis and results interpretation. MZ, BZ, L-QL, and X-NF revised and edited the manuscript. All authors contributed to the article and approved the submitted version.

## Supplementary material

Supplementary Figure 1 The flow chart of this study.
