# Supplementary figures and images for: Treatment and survival analysis for 40-year SEER data on upper esophageal cancer
Source: Front Med (Lausanne). 2023 Jul 17;10:1128766. doi: 10.3389/fmed.2023.1128766 (PMC10387539; doi:10.3389/fmed.2023.1128766)

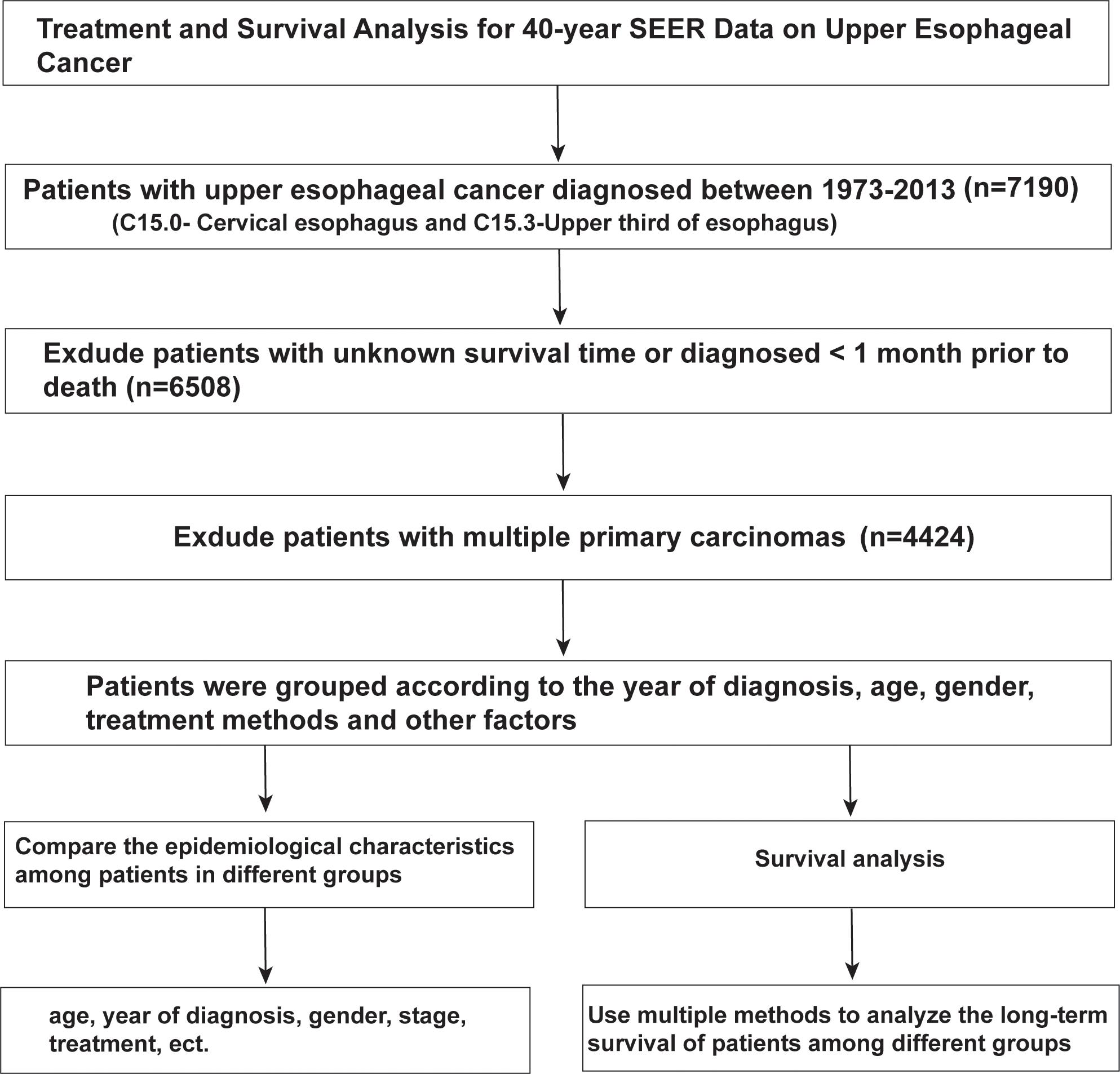

Supplement: Supplementary Figure 1 — The flow chart of this study. [file Image_1.TIF]
