# Supplementary material for: Treatment and survival analysis for 40-year SEER data on upper esophageal cancer
Source: Front Med (Lausanne). 2023 Jul 17;10:1128766. doi: 10.3389/fmed.2023.1128766 (PMC10387539; doi:10.3389/fmed.2023.1128766)
Supplement: Supplementary file 2 [file Table_1.DOCX]

Table 1: Characteristics of patients with upper esophagus cancer.

| **Characteristics** | **Number** |
| --- | --- |
| **Age** (Year)  ≤45  >45, ≤65  >65, ≤80  >80 | 66.8±11.5  130 (2.9%)  1868 (42.2%)  1900 (43.0%)  526 (11.9%) |
| **Year of diagnosis**  1973~1983  1984~1993  1994~2003  2004~2013 | 784 (17.7%)  837 (18.9%)  1197 (27.1%)  1606 (36.3%) |
| **Gender**  Male  Female | 2944 (66.5%)  1480 (33.5%) |
| **Ethnicity**  White  Black  Others  Unknown | 3092 (70.1%)  949 (21.5%)  370 (8.4%)  13 |
| **Marital Status**  Married  Single (unmarried)  Separated/Divorced/Widowed  Unknown | 2103 (49.5%)  736 (17.3%)  1410 (33.2%)  175 |
| **Grade**  Well differentiated  Moderate differentiated  Poor differentiated  Undifferentiated  Unknown | 270 (8.1%)  1485 (44.5%)  1487 (44.5%)  96 (2.9%)  1086 |
| **SEER historic stage**  Localized  Regional  Distant  Unstaged | 1114 (31.0%)  1353 (37.6%)  1131 (31.4%)  826 |
| **Histologic subtype**  Squamous  Adenocarcinoma  Others | 3775 (85.3%)  372 (8.4%)  277 (6.3%) |
| **Surgery**  Yes  No  Unknown | 560 (13.3%)  3639 (86.7%)  225 |
| **Radiation**  Yes  No  Unknown | 3291 (75.7%)  1055 (24.3%)  78 |

Table 2: Patient characteristics by year of diagnosis.

|  | 1973-1983 | 1984-1993 | 1994-2003 | 2004-2013 | p Value |
| --- | --- | --- | --- | --- | --- |
| Age |  |  |  |  | < 0.001 |
| ≤45 | 21 (2.7%) | 24 (2.9%) | 40 (3.3%) | 45 (2.8%) |  |
| >45, ≤65 | 382 (48.7%) | 328 (39.2%) | 437 (36.5%) | 721 (44.9%) |  |
| >65, ≤80 | 325 (41.5%) | 395 (47.2%) | 547 (45.7%) | 633 (39.4%) |  |
| >80 | 56 (7.1%) | 90 (10.8%) | 173 (14.5%) | 207 (12.9%) |  |
| **Gender** |  |  |  |  | 0.121 |
| Male | 535 (68.2%) | 548 (65.5%) | 769 (64.2%) | 1092 (68.0%) |  |
| Female | 249 (31.8%) | 289 (34.5%) | 428 (35.8%) | 514 (32.0%) |  |
| **Ethnicity** |  |  |  |  | < 0.001 |
| White | 595 (75.9%) | 584 (69.9%) | 810 (67.8%) | 1103 (69.1%) |  |
| Black | 141 (18.0%) | 199 (23.8%) | 274 (22.9%) | 335 (21.0%) |  |
| Other | 48 (6.1%) | 53 (6.3%) | 111 (9.3%) | 158 (9.9%) |  |
| Unknown | 0 | 1 | 2 | 10 |  |
| **Marital status** |  |  |  |  | < 0.001 |
| Married | 411 (54.5%) | 424 (52.7%) | 555 (47.9%) | 713 (46.5%) |  |
| Single (unmarried) | 89 (11.8%) | 94 (11.7%) | 208 (17.9%) | 345 (22.5%) |  |
| Separated/Divorced/Widowed | 254 (33.7%) | 286 (35.6%) | 396 (34.2%) | 474 (30.9%) |  |
| Unknown | 30 | 33 | 38 | 74 |  |
| **Histologic subtype** |  |  |  |  | < 0.001 |
| Squamous | 691 (88.1%) | 739 (88.3%) | 1012 (84.5%) | 1333 (83.0%) |  |
| Adenocarcinoma | 28 (3.6%) | 43 (5.1%) | 111 (9.3%) | 190 (11.8%) |  |
| Others | 65 (8.3%) | 55 (6.6%) | 74 (6.2%) | 83 (5.2%) |  |
| **Grade** |  |  |  |  | < 0.001 |
| Well differentiated | 74 (15.0%) | 56 (8.7%) | 67 (7.1%) | 73 (5.8%) |  |
| Moderately differentiated | 184 (37.2%) | 263 (41.0%) | 417 (44.0%) | 621 (49.5%) |  |
| Poorly differentiated | 199 (40.3%) | 300 (46.8%) | 446 (47.0%) | 542 (43.2%) |  |
| Undifferentiated | 37 (7.5%) | 22 (3.4%) | 18 (1.9%) | 19 (1.5%) |  |
| Unknown | 290 | 196 | 249 | 351 |  |
| **SEER historic stage** |  |  |  |  | < 0.001 |
| Localized | 267 (47.7%) | 230 (36.9%) | 293 (29.4%) | 324 (22.8%) |  |
| Regional | 133 (23.8%) | 230 (36.9%) | 432 (43.4%) | 558 (39.3%) |  |
| Distant | 160 (28.6%) | 163 (26.2%) | 270 (27.1%) | 538 (37.9%) |  |
| Unstaged | 224 | 214 | 202 | 186 |  |
| **Surgery** |  |  |  |  | < 0.001 |
| Yes | 74 (11.8%) | 147 (18.8%) | 186 (15.6%) | 153 (9.6%) |  |
| No | 554 (88.2%) | 633 (81.2%) | 1009 (84.4%) | 1443 (90.4%) |  |
| Unknown | 156 | 57 | 2 | 10 |  |
| **Radiation** |  |  |  |  | < 0.001 |
| Yes | 658 (85.1%) | 657 (80.0%) | 879 (75.1%) | 1097 (69.4%) |  |
| No | 115 (14.9%) | 164 (20.0%) | 292 (24.9%) | 484 (30.6%) |  |
| Unknown | 11 | 16 | 26 | 25 |  |

Table 3: Patient characteristics across SEER historic stages.

|  | Localized | Regional | Distant | p Value |
| --- | --- | --- | --- | --- |
| **Year of diagnosis** |  |  |  | < 0.001 |
| 1973~1983 | 267 (24.0%) | 133 (9.8%) | 160 (14.1%) |  |
| 1984~1993 | 230 (20.6%) | 230 (17.0%) | 163 (14.4%) |  |
| 1994-2003 | 293 (26.3%) | 432 (31.9%) | 270 (23.9%) |  |
| 2004-2013 | 324 (29.1%) | 558 (41.2%) | 538 (47.6%) |  |
| Age |  |  |  | < 0.001 |
| ≤45 | 27 (2.4%) | 49 (3.6%) | 36 (3.2%) |  |
| >45, ≤65 | 407 (36.5%) | 615 (45.5%) | 574 (50.8%) |  |
| >65, ≤80 | 514 (46.1%) | 567 (41.9%) | 434 (38.4%) |  |
| >80 | 166 (14.9%) | 122 (9.0%) | 87 (7.7%) |  |
| **Gender** |  |  |  | < 0.001 |
| Male | 713 (64.0%) | 915 (67.6%) | 811 (71.7%) |  |
| Female | 401 (36.0%) | 438 (32.4%) | 320 (28.3%) |  |
| **Ethnicity** |  |  |  | < 0.001 |
| White | 819 (73.6%) | 961 (71.1%) | 715 (63.3%) |  |
| Black | 216 (19.4%) | 269 (19.9%) | 313 (27.7%) |  |
| Other | 78 (7.0%) | 121 (9.0%) | 101 (8.9%) |  |
| Unknown | 1 | 2 | 5 |  |
| **Marital status** |  |  |  | 0.007 |
| Married | 552 (51.8%) | 658 (50.4%) | 523 (47.8%) |  |
| Single (unmarried) | 156 (14.6%) | 237 (18.2%) | 227 (20.8%) |  |
| Separated/Divorced/Widowed | 358 (33.6%) | 410 (31.4%) | 343 (31.4%) |  |
| Unknown | 48 | 48 | 38 |  |
| **Histologic subtype** |  |  |  | < 0.001 |
| Squamous | 937 (84.1%) | 1198 (88.5%) | 946 (83.6%) |  |
| Adenocarcinoma | 119 (10.7%) | 88 (6.5%) | 107 (9.5%) |  |
| Others | 58 (5.2%) | 67 (5.0%) | 78 (6.9%) |  |
| **Grade** |  |  |  | 0.001 |
| Well differentiated | 93 (11.0%) | 81 (7.5%) | 53 (6.3%) |  |
| Moderately differentiated | 377 (44.8%) | 499 (46.5%) | 347 (41.3%) |  |
| Poorly differentiated | 350 (41.6%) | 469 (43.7%) | 411 (48.9%) |  |
| Undifferentiated | 22 (2.6%) | 24 (2.2%) | 29 (3.5%) |  |
| Unknown | 272 | 280 | 291 |  |
| **Surgery** |  |  |  | < 0.001 |
| Yes | 191 (18.9%) | 240 (18.2%) | 80 (7.4%) |  |
| No | 821 (81.1%) | 1079 (81.8%) | 1005 (92.6%) |  |
| Unknown | 102 | 34 | 46 |  |
| **Radiation** |  |  |  | < 0.001 |
| Yes | 820 (75.0%) | 1090 (81.8%) | 826 (74.1%) |  |
| No | 274 (25.0%) | 242 (18.2%) | 288 (25.9%) |  |
| Unknown | 20 | 21 | 17 |  |

Table 4: Patients characteristics across histologic subtypes.

|  | SCC | AC | Other types | P value |
| --- | --- | --- | --- | --- |
| Year of diagnosis |  |  |  | p < 0.001 |
| 1973~1983 | 691 (18.3%) | 28 (7.5%) | 65 (23.5%) |  |
| 1984~1993 | 739 (19.6%) | 43 (11.6%) | 55 (19.9%) |  |
| 1994~2003 | 1012 (26.8%) | 111 (29.8%) | 74 (26.7%) |  |
| 2004~2014 | 1333 (35.3%) | 190 (51.1%) | 83 (30.0%) |  |
| Ethnicity |  |  |  | p < 0.001 |
| White | 2538 (67.4%) | 337 (90.8%) | 217 (78.3%) |  |
| Black | 882 (23.4%) | 20 (5.4%) | 47 (17.0%) |  |
| Other | 343 (9.1%) | 14 (3.8%) | 13 (4.7%) |  |
| Unknown | 12 | 1 | 0 |  |
| Sex |  |  |  | p < 0.001 |
| Male | 2489 (65.9%) | 285 (76.6%) | 170 (61.4%) |  |
| Female | 1286 (34.1%) | 87 (23.4%) | 107 (38.6%) |  |
| Age |  |  |  | p < 0.001 |
| ≤45 | 108 (2.9%) | 14 (3.8%) | 8 (2.9%) |  |
| >45,≤65 | 1615 (42.8%) | 165 (44.4%) | 88 (31.8%) |  |
| >65,≤80 | 1635 (43.3%) | 146 (39.2%) | 119 (43.0%) |  |
| >80 | 417 (11.0%) | 47 (12.6%) | 62 (22.4%) |  |
| Marital status |  |  |  | p = 0.009 |
| Married | 1769 (48.8%) | 201 (56.6%) | 133 (49.6%) |  |
| Single (unmarried) | 638 (17.6%) | 62 (17.5%) | 36 (13.4%) |  |
| Separated/Divorced/Widowed | 1219 (33.6%) | 92 (25.9%) | 99 (36.9%) |  |
| Unknown | 149 | 17 | 9 |  |
| Grade |  |  |  | p < 0.001 |
| Well differentiated | 242 (8.3%) | 25 (8.5%) | 3 (2.3%) |  |
| Moderately differentiated | 1388 (47.6%) | 95 (32.4%) | 2 (1.6%) |  |
| Poorly differentiated | 1237 (42.4%) | 168 (57.3%) | 82 (63.6%) |  |
| Undifferentiated | 49 (1.7%) | 5 (1.7%) | 42 (32.6%) |  |
| Unknown | 859 | 79 | 148 |  |
| SEER historic stage |  |  |  | p < 0.001 |
| Localized | 937 (30.4%) | 119 (37.9%) | 58 (28.6%) |  |
| Regional | 1198 (38.9%) | 88 (28.0%) | 67 (33.0%) |  |
| Distant | 946 (30.7%) | 107 (34.1%) | 78 (38.4%) |  |
| Unstaged | 694 | 58 | 74 |  |
| Surgery |  |  |  | p < 0.001 |
| Yes | 447 (12.5%) | 88 (24.3%) | 25 (9.9%) |  |
| No | 3137 (87.5%) | 274 (75.7%) | 228 (90.1%) |  |
| Unknown | 191 | 10 | 24 |  |
| Radiation |  |  |  | p < 0.001 |
| Yes | 2932 (79.1%) | 200 (54.6%) | 159 (58.5%) |  |
| No | 776 (20.9%) | 166 (45.4%) | 113 (41.5%) |  |
| Unknown | 67 | 6 | 5 |  |

Table 5: Patient characteristics across treatments.

|  | None | RWS | SWR | R+S | p Value |
| --- | --- | --- | --- | --- | --- |
| **Year of diagnosis** |  |  |  |  | < 0.001 |
| 1973~1983 | 63 (8.0%) | 482 (17.3%) | 38 (16.0%) | 36 (11.2%) |  |
| 1984~1993 | 96 (12.2%) | 525 (18.9%) | 57 (23.9%) | 90 (28.0%) |  |
| 1994~2003 | 220 (27.9%) | 763 (27.4%) | 72 (30.3%) | 114 (35.4%) |  |
| 2004~2013 | 409 (51.9%) | 1013 (36.4%) | 71 (29.8%) | 82 (25.5%) |  |
| **Ethnicity** |  |  |  |  | 0.073 |
| White | 528 (67.5%) | 1913 (68.9%) | 184 (77.3%) | 229 (71.1%) |  |
| Black | 191 (24.4%) | 611 (22.0%) | 37 (15.5%) | 64 (19.9%) |  |
| Other | 63 (8.1%) | 252 (9.1%) | 17 (7.2%) | 29 (9.0%) |  |
| Unknown | 6 | 7 | 0 | 0 |  |
| **Gender** |  |  |  |  | 0.491 |
| Male | 510 (64.7%) | 1846 (66.3%) | 158 (66.4%) | 224 (69.6%) |  |
| Female | 278 (35.3%) | 937 (33.7%) | 80 (33.6%) | 98 (30.4%) |  |
| **Age** |  |  |  |  | < 0.001 |
| ≤45 | 22 (2.8%) | 72 (2.6%) | 10 (4.2%) | 18 (5.6%) |  |
| >45, ≤65 | 267 (33.9%) | 1196 (43.0%) | 113 (47.5%) | 167 (51.9%) |  |
| >65, ≤80 | 326 (41.4%) | 1225 (44.0%) | 96 (40.3%) | 126 (39.1%) |  |
| >80 | 173 (22.0%) | 290 (10.4%) | 19 (8.0%) | 11 (3.4%) |  |
| **Marital status** |  |  |  |  | < 0.001 |
| Married | 298 (40.7%) | 1336 (49.5%) | 138 (60.0%) | 189 (59.6%) |  |
| Single (unmarried) | 169 (23.1%) | 453 (16.8%) | 37 (16.1%) | 46 (14.5%) |  |
| Separated/Divorced/Widowed | 266 (36.3%) | 909 (33.7%) | 55 (23.9%) | 82 (25.9%) |  |
| Unknown | 55 | 85 | 8 | 5 |  |
| **Histologic subtype** |  |  |  |  | < 0.001 |
| Squamous | 591 (75.0%) | 2487 (89.4%) | 163 (68.5%) | 284 (88.2%) |  |
| Adenocarcinoma | 101 (12.8%) | 168 (6.0%) | 62 (26.1%) | 26 (8.1%) |  |
| Others | 96 (12.2%) | 128 (4.6%) | 13 (5.5%) | 12 (3.7%) |  |
| **Grade** |  |  |  |  | < 0.001 |
| Well differentiated | 24 (4.2%) | 176 (8.3%) | 26 (13.9%) | 20 (7.5%) |  |
| Moderately differentiated | 224 (39.6%) | 962 (45.4%) | 87 (46.5%) | 119 (44.9%) |  |
| Poorly differentiated | 296 (52.3%) | 927 (43.7%) | 67 (35.8%) | 123 (46.4%) |  |
| Undifferentiated | 22 (3.9%) | 54 (2.5%) | 7 (3.7%) | 3 (1.1%) |  |
| Unknown | 222 | 664 | 51 | 57 |  |
| **SEER historic stage** |  |  |  |  | < 0.001 |
| Localized | 155 (27.5%) | 649 (28.4%) | 109 (49.3%) | 82 (28.3%) |  |
| Regional | 154 (27.3%) | 905 (39.6%) | 85 (38.5%) | 155 (53.4%) |  |
| Distant | 255 (45.2%) | 734 (32.6%) | 27 (12.2%) | 53 (18.3%) |  |
| Unstaged | 224 | 495 | 17 | 32 |  |

Table 6: Patient characteristics across radiation sequences.

|  | **SWR** | **NRT** | **ART** | **p Value** |
| --- | --- | --- | --- | --- |
| **Year of diagnosis** |  |  |  | 0.022 |
| 1973~1983 | 38 (16.0%) | 16 (12.0%) | 17 (9.7%) |  |
| 1984~1993 | 57 (23.9%) | 32 (24.1%) | 56 (32.0%) |  |
| 1994~2003 | 72 (30.3%) | 41 (30.8%) | 68 (38.9%) |  |
| 2004~2013 | 71 (29.8%) | 44 (33.1%) | 34 (19.4%) |  |
| **Ethnicity** |  |  |  | 0.682 |
| White | 184 (77.3%) | 95 (71.4%) | 128 (73.1%) |  |
| Black | 37 (15.5%) | 25 (18.8%) | 34 (19.5%) |  |
| Other | 17 (7.1%) | 13 (9.8%) | 13 (7.4%) |  |
| **Gender** |  |  |  | 0.781 |
| Male | 158 (66.4%) | 93 (69.9%) | 119 (68.0%) |  |
| Female | 80 (33.6%) | 40 (30.1%) | 56 (32.0%) |  |
| **Age** |  |  |  | 0.125 |
| ≤45 | 10 (4.2%) | 6 (4.5%) | 11 (6.3%) |  |
| >45, ≤65 | 113 (47.5%) | 76 (57.1%) | 82 (46.9%) |  |
| >65, ≤80 | 96 (40.3%) | 49 (36.8%) | 73 (41.7%) |  |
| >80 | 19 (8.0%) | 2 (1.5%) | 9 (5.1%) |  |
| **Marital status** |  |  |  | 0.139 |
| Married | 138 (60.0%) | 86 (66.2%) | 98 (56.0%) |  |
| Single (unmarried) | 37 (16.1%) | 11 (8.5%) | 33 (18.9%) |  |
| Separated/Divorced/Widowed | 55 (23.9%) | 33 (25.4%) | 44 (25.1%) |  |
| Unknown | 8 | 3 | 0 |  |
| **Histologic subtype** |  |  |  | < 0.001 |
| Squamous | 163 (68.5%) | 119 (89.5%) | 152 (86.9%) |  |
| Adenocarcinoma | 62 (26.1%) | 11 (8.3%) | 14 (8.0%) |  |
| Others | 13 (5.5%) | 3 (2.3%) | 9 (5.1%) |  |
| **Grade** |  |  |  | 0.035 |
| Well differentiated | 26 (13.9%) | 5 (4.7%) | 14 (9.4%) |  |
| Moderately differentiated | 87 (46.5%) | 51 (48.1%) | 63 (42.3%) |  |
| Poorly differentiated | 67 (35.8%) | 50 (47.2%) | 69 (46.3%) |  |
| Undifferentiated | 7 (3.7%) | 0 | 3 (2.0%) |  |
| Unknown | 51 | 27 | 26 |  |
| **SEER historic stage** |  |  |  | < 0.001 |
| Localized | 109 (49.3%) | 36 (30.8%) | 41 (25.8%) |  |
| Regional | 85 (38.5%) | 58 (49.6%) | 92 (57.9%) |  |
| Distant | 27 (11.3%) | 23 (19.7%) | 26 (16.4%) |  |
| Unstaged | 17 | 16 | 16 |  |

Table 7: Univariate analysis of ECSS and OS of all UEC patients.

| Variable | ECSS | | | OS | | |
| --- | --- | --- | --- | --- | --- | --- |
|  | HR | 95% CI of HR | P value | HR | 95% CI of HR | P value |
| **Year of diagnosis** |  |  |  |  |  |  |
| 1973~1983 | Reference |  |  | Reference |  |  |
| 1984~1993 | 0.804 | 0.724 – 0.894 | < 0.001 | 0.818 | 0.742 – 0.903 | < 0.001 |
| 1994~2003 | 0.750 | 0.680 – 0.827 | < 0.001 | 0.745 | 0.680 – 0.816 | < 0.001 |
| 2004~2013 | 0.636 | 0.578 – 0.700 | < 0.001 | 0.629 | 0.574 – 0.688 | < 0.001 |
| **Ethnicity** |  |  |  |  |  |  |
| White | Reference |  |  | Reference |  |  |
| Black | 1.233 | 1.137 – 1.337 | < 0.001 | 1.212 | 1.123 – 1.307 | < 0.001 |
| Other | 0.419 | 0.837 – 1.077 | 0.419 | 0.924 | 0.821 – 1.040 | 0.188 |
| Unknown | 1.287 | 0.659 – 2.440 | 0.478 | 1.140 | 0.592 – 2.193 | 0.696 |
| **Sex** (Male) |  |  |  |  |  |  |
| Male | Reference |  |  | Reference |  |  |
| Female | 0.836 | 0.878 – 0.943 | < 0.001 | 0.880 | 0.823 – 0.940 | < 0.001 |
| **Age** |  |  |  |  |  |  |
| ≤45 | Reference |  |  | Reference |  |  |
| >45,≤65 | 1.583 | 1.289 – 1.943 | < 0.001 | 1.564 | 1.277 – 1.917 | < 0.001 |
| >65,≤80 | 1.726 | 1.405 – 2.121 | < 0.001 | 1.637 | 1.336 – 2.006 | < 0.001 |
| >80 | 2.107 | 1.687 – 2.631 | < 0.001 | 1.956 | 1.574 – 2.430 | < 0.001 |
| **Marital status** |  |  |  |  |  |  |
| Married | Reference |  |  | Reference |  |  |
| Single (unmarried) | 1.116 | 1.017 – 1.226 | 0.021 | 1.111 | 1.015 – 1.215 | 0.022 |
| Separated/Divorced  /Widowed | 1.129 | 1.047 – 1.216 | 0.001 | 1.165 | 1.085 – 1.250 | < 0.001 |
| Unknown | 1.098 | 0.932 – 1.294 | 0.265 | 1.170 | 0.995 – 1.375 | 0.057 |
| **Histologic subtype** |  |  |  |  |  |  |
| Squamous | Reference |  |  | Reference |  |  |
| Adenocarcinoma | 0.766 | 0.675 – 0.870 | < 0.001 | 0.769 | 0.682 – 0.867 | < 0.001 |
| Others | 1.241 | 1.083 –1.422 | 0.002 | 1.278 | 1.120 – 1.459 | < 0.001 |
| **Grade** |  |  |  |  |  |  |
| Well differentiated | Reference |  |  | Reference |  |  |
| Moderately differentiated | 1.093 | 0.942 – 1.268 | 0.241 | 1.099 | 0.957 – 1.262 | 0.180 |
| Poorly differentiated | 1.196 | 1.031 – 1.387 | 0.018 | 1.221 | 1.064 – 1.401 | 0.005 |
| Undifferentiated | 1.610 | 1.248 – 2.078 | < 0.001 | 1.635 | 1.286 – 2.078 | < 0.001 |
| Unknown | 1.233 | 1.059 – 1.436 | 0.007 | 1.253 | 1.088 – 1.444 | 0.002 |
| **SEER historic stage** |  |  |  |  |  |  |
| Localized | Reference |  |  | Reference |  |  |
| Regional | 1.295 | 1.180 – 1.420 | < 0.001 | 1.229 | 1.129 – 1.338 | < 0.001 |
| Distant | 2.167 | 1.971 – 2.382 | < 0.001 | 1.961 | 1.795 – 2.143 | < 0.001 |
| Unstaged | 1.469 | 1.324 – 1.629 | < 0.001 | 1.443 | 1.313 – 1.586 | < 0.001 |
| **Surgery** |  |  |  |  |  |  |
| No | Reference |  |  | Reference |  |  |
| Yes | 0.599 | 0.538 – 0.666 | < 0.001 | 0.617 | 0.560 – 0.680 | < 0.001 |
| Unknown | 1.218 | 1.055 – 1.407 | 0.007 | 1.211 | 1.057 – 1.387 | 0.006 |
| **Radiation** |  |  |  |  |  |  |
| No | Reference |  |  | Reference |  |  |
| Yes | 0.749 | 0.692 – 0.811 | < 0.001 | 0.736 | 0.684 – 0.792 | < 0.001 |
| Unknown | 1.088 | 0.842 – 1.406 | 0.518 | 1.032 | 0.810 – 1.313 | 0.801 |

Table 8: Multivariate analysis of ECSS and OS of all UEC patients.

| Variable | **ECSS** | | | **OS** | | |
| --- | --- | --- | --- | --- | --- | --- |
|  | HR | 95% CI of HR | P value | HR | 95% CI of HR | P value |
| **Year of diagnosis** |  |  |  |  |  |  |
| 1973~1983 | Reference |  |  | Reference |  |  |
| 1984~1993 | 0.777 | 0.698 – 0.866 | < 0.001 | 0.791 | 0.715 – 0.875 | < 0.001 |
| 1994~2003 | 0.679 | 0.610 – 0.755 | < 0.001 | 0.677 | 0.613 – 0.747 | < 0.001 |
| 2004~2013 | 0.505 | 0.453 – 0.562 | < 0.001 | 0.509 | 0.460 – 0.563 | < 0.001 |
| **Ethnicity** |  |  |  |  |  |  |
| White | Reference |  |  | Reference |  |  |
| Black | 1.158 | 1.063 – 1.261 | 0.001 | 1.165 | 1.075 – 1.262 | < 0.001 |
| Other | 0.967 | 0.852 – 1.099 | 0.611 | 0.945 | 0.838 – 1.065 | 0.350 |
| Unknown | 1.399 | 0.724 – 2.704 | 0.317 | 1.242 | 0.643 – 2.397 | 0.519 |
| **Sex (Male)** |  |  |  |  |  |  |
| Male | Reference |  |  | Reference |  |  |
| Female | 0.844 | 0.783 – 0.910 | < 0.001 | 0.835 | 0.779 – 0.895 | < 0.001 |
| **Age** |  |  |  |  |  |  |
| ≤45 | Reference |  |  | Reference |  |  |
| >45,≤65 | 1.505 | 1.214 – 1.865 | < 0.001 | 1.606 | 1.309 – 1.971 | < 0.001 |
| >65,≤80 | 1.531 | 1.234 – 1.899 | < 0.001 | 1.733 | 1.411 – 2.128 | < 0.001 |
| >80 | 1.844 | 1.459 – 2.330 | < 0.001 | 2.139 | 1.714 – 2.669 | < 0.001 |
| **Marital status** |  |  |  |  |  |  |
| Married | Reference |  |  | Reference |  |  |
| Single (unmarried) | 1.131 | 1.024 – 1.250 | 0.015 | 1.131 | 1.030 – 1.242 | 0.010 |
| Separated/Divorced  /Widowed | 1.147 | 1.058 – 1.242 | 0.001 | 1.132 | 1.050 – 1.220 | 0.001 |
| Unknown | 1.176 | 0.989 – 1.398 | 0.066 | 1.111 | 0.943 – 1.308 | 0.210 |
| **Histologic subtype** |  |  |  |  |  |  |
| Squamous | Reference |  |  | Reference |  |  |
| Adenocarcinoma | 0863 | 0.756 – 0.985 | 0.029 | 0.857 | 0.758 – 0.969 | 0.014 |
| Others | 1.066 | 0.928 – 1.225 | 0.364 | 1.083 | 0.953 –1.230 | 0.222 |
| **SEER historic stage** |  |  |  |  |  |  |
| Localized | Reference |  |  | Reference |  |  |
| Regional | 1.518 | 1.380 – 1.670 | < 0.001 | 1.450 | 1.328 – 1.583 | < 0.001 |
| Distant | 2.336 | 2.116 – 2.579 | < 0.001 | 2.135 | 1.947 – 2.342 | < 0.001 |
| Unstaged | 1.258 | 1.131 – 1.399 | < 0.001 | 1.235 | 1.121 – 1.361 | < 0.001 |
| **Surgery** |  |  |  |  |  |  |
| No | Reference |  |  | Reference |  |  |
| Yes | 0.563 | 0.502 – 0.631 | < 0.001 | 0.578 | 0.521 – 0.643 | < 0.001 |
| Unknown | 1.067 | 0.914 –1.245 | 0.412 | 1.050 | 0.908 – 1.215 | 0.509 |
| **Radiation** |  |  |  |  |  |  |
| No | Reference |  |  | Reference |  |  |
| Yes | 0.579 | 0.531 – 0.631 | < 0.001 | 0.569 | 0.525 – 0.617 | < 0.001 |
| Unknown | 0.839 | 0.647 –1.087 | 0.185 | 0.788 | 0.617 – 1.006 | 0.056 |

Table 9: Univariate analysis of ECSS and OS across treatments.

| Variable | ECSS | | | OS | | |
| --- | --- | --- | --- | --- | --- | --- |
|  | HR | 95% CI of HR | P value | HR | 95% CI of HR | P value |
| Treatment |  |  |  |  |  |  |
| None | Reference |  |  | Reference |  |  |
| RWS | 0.560 | 0.513 – 0.613 | < 0.001 | 0.548 | 0.504 – 0.596 | < 0.001 |
| SWR | 0.344 | 0.288 – 0.412 | < 0.001 | 0.351 | 0.299 – 0.413 | < 0.001 |
| R+S | 0.392 | 0.337 – 0.456 | < 0.001 | 0.394 | 0.343 – 0.452 | < 0.001 |
| **Year of diagnosis** |  |  |  |  |  |  |
| 1973~1983 | Reference |  |  | Reference |  |  |
| 1984~1993 | 0.811 | 0.723 – 0.910 | < 0.001 | 0.818 | 0.742 – 0.903 | < 0.001 |
| 1994~2003 | 0. 758 | 0.682 – 0.843 | < 0.001 | 0.745 | 0.680 – 0.816 | < 0.001 |
| 2004~2013 | 0.639 | 0.576 – 0.709 | < 0.001 | 0.629 | 0.574 – 0.688 | < 0.001 |
| **Ethnicity** |  |  |  |  |  |  |
| White | Reference |  |  | Reference |  |  |
| Black | 1.248 | 1.148 – 1.356 | < 0.001 | 1.212 | 1.123 – 1.307 | < 0.001 |
| Other | 0.984 | 0.866 – 1.118 | 0.800 | 0.924 | 0.821 – 1.040 | 0.188 |
| Unknown | 0.432 | 1.300 – 2.502 | 0.432 | 1.140 | 0.592 – 2.193 | 0.696 |
| **Sex** (Male) |  |  |  |  |  |  |
| Male | Reference |  |  | Reference |  |  |
| Female | 0.874 | 0.811 – 0.941 | < 0.001 | 0.880 | 0.823 – 0.940 | < 0.001 |
| **Age** |  |  |  |  |  |  |
| ≤45 | Reference |  |  | Reference |  |  |
| >45,≤65 | 1.480 | 1.187 – 1.845 | < 0.001 | 1.564 | 1.277 – 1.917 | < 0.001 |
| >65,≤80 | 1.468 | 1.177 – 1.830 | 0.001 | 1.637 | 1.336 – 2.006 | < 0.001 |
| >80 | 1.662 | 1.311 – 2.107 | < 0.001 | 1.956 | 1.574 – 2.430 | < 0.001 |
| **Marital status** |  |  |  |  |  |  |
| Married | Reference |  |  | Reference |  |  |
| Single (unmarried) | 1.149 | 1.041 – 1.268 | 0.006 | 1.111 | 1.015 – 1.215 | 0.022 |
| Separated/Divorced  /Widowed | 1.169 | 1.080 –1.266 | 0.001 | 1.165 | 1.085 – 1.250 | < 0.001 |
| Unknown | 1.198 | 0.998 – 1.438 | 0.052 | 1.170 | 0.995 – 1.375 | 0.057 |
| **Histologic subtype** |  |  |  |  |  |  |
| Squamous | Reference |  |  | Reference |  |  |
| Adenocarcinoma | 0.773 | 0.679 – 0.880 | < 0.001 | 0.769 | 0.682 – 0.867 | < 0.001 |
| Others | 1.246 | 1.079 – 1.439 | 0.003 | 1.278 | 1.120 – 1.459 | < 0.001 |
| **Grade** |  |  |  |  |  |  |
| Well differentiated | Reference |  |  | Reference |  |  |
| Moderately differentiated | 1.103 | 0.944 – 1.288 | 0.219 | 1.099 | 0.957 – 1.262 | 0.180 |
| Poorly differentiated | 1.221 | 1.046 – 1.426 | 0.012 | 1.221 | 1.064 – 1.401 | 0.005 |
| Undifferentiated | 1.638 | 1.251 – 2.144 | < 0.001 | 1.635 | 1.286 – 2.078 | < 0.001 |
| Unknown | 1.261 | 1.075 – 1.480 | 0.004 | 1.253 | 1.088 – 1.444 | 0.002 |
| **SEER historic stage** |  |  |  |  |  |  |
| Localized | Reference |  |  | Reference |  |  |
| Regional | 1.335 | 1.212 – 1.472 | < 0.001 | 1.229 | 1.129 – 1.338 | < 0.001 |
| Distant | 2.217 | 2.006 – 2.449 | < 0.001 | 1.961 | 1.795 – 2.143 | < 0.001 |
| Unstaged | 1.536 | 1.377 – 1.713 | < 0.001 | 1.443 | 1.313 – 1.586 | < 0.001 |

**Table 10:** Univariate and Multivariate analysis of OS across radiation sequences for SCC in regional stage.

| Variable | Univariate analysis | | | Multivariate analysis | | |
| --- | --- | --- | --- | --- | --- | --- |
|  | HR | 95% CI of HR | P value | HR | 95% CI of HR | P value |
| Group |  |  |  |  |  |  |
| SWR | Reference |  |  | Reference |  |  |
| NRT | 0.633 | 0.427 – 0.938 | 0.023 | 0.633 | 0.427 – 0.938 | 0.023 |
| ART | 0.635 | 0.453 – 0.889 | 0.008 | 0.635 | 0.453 – 0.889 | 0.008 |
| **Year of diagnosis** |  |  |  |  |  |  |
| 1973~1983 | Reference |  |  | Reference |  |  |
| 1984~1993 | 0.861 | 0.541 – 1.372 | 0.529 | --- | --- | --- |
| 1994~2003 | 0.595 | 0. 376 – 0. 941 | 0.026 | --- | --- | --- |
| 2004~2013 | 0.608 | 0. 370 –1.000 | 0.050 | --- | --- | --- |
| **Ethnicity** |  |  |  |  |  |  |
| White | Reference |  |  | Reference |  |  |
| Black | 1.269 | 0.892 – 1.807 | 0.186 | --- | --- | --- |
| Other | 0.660 | 0.346 – 1.256 | 0.206 | --- | --- | --- |
| **Sex (Male)** |  |  |  |  |  |  |
| Male | Reference |  |  | Reference |  |  |
| Female | 0.785 | 0.572 - 1.076 | 0.132 | --- | --- | --- |
| **Grade** |  |  |  |  |  |  |
| Well differentiated | Reference |  |  | Reference |  |  |
| Moderately differentiated | 0.953 | 0.528 – 1.722 | 0.874 | --- | --- | --- |
| Poorly differentiated | 0.877 | 0.482 – 1.598 | 0.669 | --- | --- | --- |
| Undifferentiated | 2.231 | 0.289 – 17.241 | 0.442 | --- | --- | --- |
| Unknown | 1.912 | 0.955 – 3.828 | 0.067 | --- | --- | --- |
| **Age** |  |  |  |  |  |  |
| ≤45 | Reference |  |  | Reference |  |  |
| >45,≤65 | 1.455 | 0.730 –2.898 | 0.286 | --- | --- | --- |
| >65,≤80 | 1.960 | 0.976 – 3.936 | 0.059 | --- | --- | --- |
| >80 | 1.427 | 0.565 – 3.604 | 0.452 | --- | --- | --- |
| **Marital status** |  |  |  |  |  |  |
| Married | Reference |  |  | Reference |  |  |
| Single (unmarried) | 1.005 | 0.662 –1.524 | 0.982 | --- | --- | --- |
| Separated/Divorced  /Widowed | 1.143 | 0.807 –1.619 | 0.450 | --- | --- | --- |
| Unknown | 2.063 | 0.650 –6.541 | 0.219 | --- | --- | --- |
